# Biomarkers for distinguishing tuberculous pleural effusion from non-tuberculosis effusion: a retrospective study

**DOI:** 10.1186/s12879-023-08781-0

**Published:** 2023-11-08

**Authors:** Guo Fei, Mo Yijun, Jin Weijiang, Chen Huimin, Liu Fang

**Affiliations:** 1 Department of Laboratory Medicine, The First Affiliated Hospital of Ningbo University, 59 Liuting Street, Haishu District, Ningbo, 315010 Zhejiang China; 2 Department of Respiratory and Critical Care Medicine, The First Affiliated Hospital of Ningbo University, Ningbo, Zhejiang China

**Keywords:** Pleural effusion, Tuberculous pleural effusion, Mycobacterium tuberculosis(Mtb), Area under the curve, Biomarkers, Ratios

## Abstract

**Background:**

Pleural effusion (PE) is a common clinical feature that presents a diagnostic challenge for clinicians. In this retrospective study, we aimed to assess the biomarkers, ratios, and multiple indicators in serum and Pleural effusion for the differential diagnosis of tuberculous pleural effusion (TPE) from non-tuberculosis effusion (non-TPE).

**Methods:**

The participants, who were divided into two groups: TPE and non-TPE (MPE and PPE), from Ningbo First Hospital, were incorporated in this study. The clinical and laboratory features were collected and analyzed using logistic regression analysis. Twelve biomarkers and their ratios in serum and PE were investigated for TPE versus non-TPE. Additionally, the value of multiple indicators for joint diagnosis was estimated.

**Results:**

Biomarkers and ratios showed good diagnostic performance. The five variables including Serum ADA, IGRA, Effusion ADA, Effusion ADA/Serum ADA and Effusion LDH/Effusion ADA were identified as valuable parameters for differential diagnosis of TPE from non-TPE. The combined diagnosis of the five indexes yielded the highest diagnostic accuracy for TPE with an AUC (0.919), sensitivity (90.30%), and specificity (94.50%).

**Conclusions:**

The biomarkers and ratios demonstrated strong diagnostic performance, and the utilization of multiple indicators for joint diagnosis can improve the diagnostic efficacy of tuberculous pleurisy.

**Supplementary Information:**

The online version contains supplementary material available at 10.1186/s12879-023-08781-0.

## Background

Tuberculosis (TB) is the second deadliest infectious disease behind the COVID-19. The World Health Organization (WHO) estimates that 10.6 million people worldwide suffered from TB in 2021, an increase of 4.5% from 10.1 million in 2020. The TB incidence rate (new cases per 100 000 population per year) rose by 3.6% between 2020 and 2021, reversing declines of about 2% per year for most of the previous 2 decades [1]. Tuberculous pleural effusion (TPE) is the second most common form of extrapulm- onary tuberculosis [2, 3], with presentations ranging from benign effusions that are absorbed spontaneously to complicated effusions with pleural thickening, empyema, and even Pleural fibrosis, all of which may result in lasting lung function impairment [4]. So, early and accurate diagnosis of TPE is extremely critical for the management of the disease.

Confirmation of TPE requires the isolation and/or culture of Mycobacterium tuberculosis (Mtb) from Pleural effusions and Pleural biopsy specimens or the demonstration of granulomas by pleural biopsy [5, 6]. In addition, the invasiveness and technical difficulty of medical thoracoscopic surgery appear to offer greater sensitivity (93–100%) and accuracy for diagnosing TPE.

However, it is an invasive and expensive diagnostic procedure with a complication rate of 2–6% [7, 8]. The common complications include bleeding, fever, empyema, pneumonia, and long-term air leakage air leak and so on [9]. Besides, some patients with advanced underlying disease progression and elderly patients could not tolerate the test.

In order to diagnose TPE, Pleural effusion and peripheral blood tests have been proposed as an alternative method [4]. These specimens are commonly used in clinical practice and are minimally invasive and easy to obtain. IGRA, CRP, ESR, serum TP, ALB, ADA, LDH, Pleural effusion TP, ALB, ADA, and LDH are the primary examinations for hospitalized patients. However, it is crucial to further investigate the application value. Therefore, we conducted a retrospective analysis.

## Materials and methods

### Study population

**Fig. 1:**
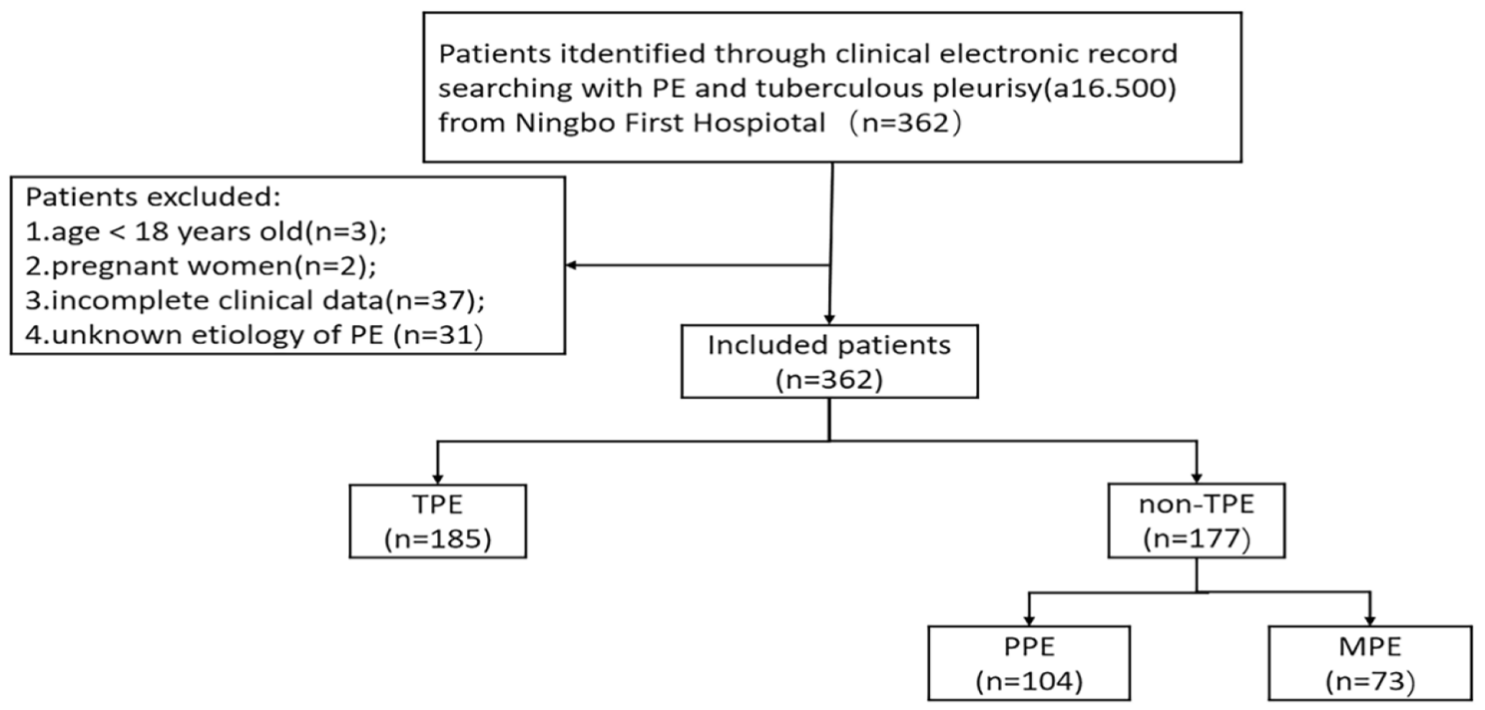
Flow diagram of study selection. PE Pleural effusion, non-TPE: non-tuberculosis effusion, TB tuberculosis

This retrospective study focused on patients newly diagnosed PE between January 2015 and March 2022 from Ningbo First Hospital. Patients under the age of 18 and those who were unwilling to provide informed consent were excluded from the study. The patient enrollment process was shown in Fig. 1.The whole patients included in the study were hospitalized for the first time owing to pleural effusion. All PE samples and followed peripheral blood samples were collected and tested. The study analyzed data from the first sample of PE and blood collected from each patient. The correlated statistics, laboratory, and clinical characteristics for all patients were obtained from the clinical electronic record system. A total of 362 patients with PE were included in this study. Of the 362 patients, 185 cases with Tuberculous pleural effusion (TPE) were diagnosed with tuberculous pleurisy effusion, 177 cases with non-TPE,104 cases were caused by parapulmonary effusion (PPE), and 73 cases with malignant Pleural effusion (MPE) were caused by primary lung cancer. All the following guidelines were included for all subjects: (i) Diagnoses of PE was experienced either ultra-sonography, chest CT, or X-ray (ii) All participants were diagnosed by cytology, thoracentesis or Pleural biopsy and follow-up (no less than 6 months). The exclusion criteria were as follows: (i) age below 18 years old; (ii) participants with incomplete clinical data;(iii)pregnant women; (iv) uncertain of the clinical diagnosis.

### Standardized diagnostic criteria for TPE, PPE, and MPE

Patients with TPE diagnosed and treated in our hospital for the first time were registered in our study, and the diagnostic criteria were: (a) The culture of pleural effusion or pleural tissue was positive for Mycobacterium tuberculosis. (b) Mycobacterium tuberculosis has been isolated from the granulomatous inflammation, which was found in pleural biopsy histology. (c)granulomatous inflamed tissue in the pleural biopsy coexisting with clinical response to antituberculosis therapy [10–12].

The diagnosis of PPE is based on: (1) Bacterial pneumonia, with no MTB in the PF obtained by continuous thoracentesis procedures and no evidence of MTB in the pathological manifestations of inflammatory pleuritis, pleural fibrosis, plaques, or chronic empyema [13]; (2) parapneumonic PE, which disappeared after anti-inflamm- atory treatment [14].

MPE was diagnosed based on: (i) The combination of cytology, thoracoscopy, and imaging studies with a minimum follow-up of 6 months. (ii) MPE was diagnosed when Pleural effusion cytology or Pleural biopsy was positive for malignant cells [15, 16].

### Data capture

All the data of clinical and laboratory, including age, gender, smoking history, effusion biochemical indexes [TP (total protein), ALB(albumin), ADA (adenosine deaminase), LDH (lactatedehy drogenase), peripheral blood indexes [CRP(C-reactive protein), ESR(erythrocyte sedimentation rate), IGRA(interferon-gamma release assay), serum indexes [TP, ALB, ADA, and LDH](Table 1), they were obtained from the clinical electronic record system.

### PE and blood indexes analysis

The subjects of the TB-IGRA experiment used dehyrogenated vacuum tubes to collect heparinized anticoagulated whole blood, and culture filter protein 10(CFP-10) and early secretory antigen 6 (ESAT-6) containing Mycobacterium tuberculosis (MTB) specific antigen were added to the test tubes. CFP-10 and ESAT-6 stimulated MTB-specific T lymphocytes to proliferate and release IFN-γ, which was detected in plasma by enzyme-linked immunoassay (Elisa). The linear range of the method is 2-400pg/ml, the value of ≤ 2 is counted as 2. The kit was provided by Wantai Biopharmaceutical Co., Ltd (Beijing, China).CRP was assayed by Immunoturbidimetry with ARISTO from Guosai Technology Co., Ltd (Shenzhen, China). ESR was assayed with Test1 provided by Italian company ALIFAX.PE and serum TP were assayed by the biuret method, ALB by the bromocresol green end point assay method, and LDH by the modified IFCC method with Olympus AU5821 of Beckman Coulter (Suzhou, China). ADA was assayed by the enzyme colorimetric method of Saike Biotechnology Co., Ltd. (Ningbo, China) with Olympus AU5821 of Beckman Colter.

### Statistical analysis

Statistical analyses were performed using SPSS 26.0 (SPSS Inc., Chicago, IL USA), and P < 0.05 was considered to be significantly different. The categorical variables were expressed as number and percentage (n, %). The continuous variables were expressed as median and interquartile range (IQR, 25–75), and analyzed by the Mann-Whitney U test. Use univariate logistic regression analysis to select the independent indicators, and the Akaike information criterion (AIC) of the multivariable logistic regression models was used to choose statistically significant variables. Expressed as estimated odds ratios (OR) and 95% confidence intervals (CI). The receiver operating characteristic (ROC) curve and the corresponding AUCs were used to evaluate the value of biomarkers to distinguish TPE from non-TPE. We also calculated sensitivity, specificity, positive predictive value (PPV), negative predictive value (NPV), positive predictive value (PLR), and negative predictive value (NLR) to measure the diagnostic accuracy.

## Results

### Participants

From January 2015 to March 2022, a total of 435 patients from Ningbo First Hospital were investigated in this study, Among them, 73 were excluded according to the exclusion criteria, including (1) age below 18 years old(n = 3); (2) pregnant women(n = 2); (3) incomplete clinical data(n = 37); (4) unknown etiology of PE (n = 31); Finally, 362 patients were included in final analysis (Fig. 1). Demographic, clinical and laboratory characteristics of the study population are summarized in Table 1.

**Table 1:** The characteristics of study participants

Characteristics	TPE	non-TPE	
		PPE	MPE
Age	47.0 (29.0–65.0)	57.0 (50.0–72.0)	68.0 (56.5–73.0)
Gender			
Female	62(33.5)	41(39.4)	17(23.3)
Male	123(66.5)	63(60.6)	56(76.9)
Smoke status			
Non-smokers	121(65.4)	61(58.6)	26(35.6)
C/F smokers	64(34.6)	43(41.4)	47(64.4)
**Effusion**			
TP (g/L)	50.6(45.9–54.2)	45.4(38.9–51.1)	47.4(36.9–51.1)
ALB(g/L)	28.6(26.0-30.9)	25.6(22.1–28.5)	26.7(18.4–30.7)
ADA(U/L)	34,2(27.7–41.9)	11.4(7.5–21.8)	7.8(6.7–14.5)
LDH(U/L)	442(299.5-607.5)	282.0(167.0-689.5)	344.0(233.0-596.0)
**Serum**			
CRP(mg/L)	44.4(17.5–79.1)	56.8(16.7-112.7)	10.3(1.6–50.1)
ESR(mm/h)	51.0(37.3–65.0)	50.0(31.3–65.8)	25.0(14.0-39.5)
TP (g/L)	67.3(62.5–72.9)	65.1(60.7–69.9)	64.4(60.6–69.9)
ALB(g/L)	35.5(32.4–38.3)	33.5(30.4–37.0)	36.2(32.7–39.3)
ADA(U/L)	11.7(8.9–14.3)	9.5(7.6–11.5)	8.8(6.5–10.6)
LDH(U/L)	184.0(160.0-208.5)	164.0(132.0-217.8)	199(157.0-262.5)
IGRA(pg/mL)	111.0(49.0-216.5)	2.5(2.0-27.5)	3.0(2.0-26.5)

Abbreviations:TP Total protein,ALB albumin ADA adenosine deaminase, LDH lactatedehy drogenase, CRP C-reactive protein, ESR erythrocyte sedimentation rate,IGRA Interferon-γ release assay

Continuous variables were presented as median and inter quartile rang (IQR, 25th–75th). Categorical variables were presented as number and percentage (n, %)

### The results of univariate and multivariate logistic regression analysis for distinguishing TPE from non-TPE

The cutoff values of those variables were determined by Youden’s indices. Supplementary file 1: Tables S1-3 showed that all of the variables were analysed by the Mann-Whitney U test between TPE and non-TPE. Additionally, the results of the univariate logistic analysis were presented in supplementary file 1: Tables S1 including 16 variables. To further investigate the diagnostic value of biomarkers, 13 variables with an AUC > 0.65 were selected for multiple regression analysis, respectively. Using the AIC method to stepwise select the regression model, resulting in the identification of the 5 most valuable variables for distinguishing TPE from non-TPE (Table 2). The results of the multivariate logistic regression analysis were summarized in Table 2, as follows: serum ADA (OR (95%CI), 0.252(0.106–0.600)), IGRA (OR (95%CI), 0.099(0.047–0.212)), effusion ADA(OR(95%CI), 0.236(0.092–0.606)), Effusion ADA/ADA(OR (95%CI), 0.186(0.066–0.524)), Effusion LDH/ ADA, (OR(95%CI), 0.242(0.113–0.520)) (Table 2).

**Table 2:** Multivariate logistic regression analysis of the clinical characteristics for discriminating TPE from non-TPE

Variables	Multivariate analysis OR (95%CI)	P value	
Serum ADA(U/l)			
< 10.95	0.252(0.106–0.600)	0.002	
≥ 10.95			
IGRA(pg/ml)			
< 26.50	0.099(0.047–0.212)	< 0.001	
≥ 26.50			
Effusion ADA(U/L)			
< 25.20	0.236(0.092–0.606)	< 0.001	
≥ 25.20			
Effusion ADA /Serum ADA			
< 2.07	0.186(0.066–0.524)	0.001	
≥ 2.07			
Effusion LDH/Effusion ADA			
< 17.49	0.242(0.113–0.520)	< 0.001	
≥ 17.49			

### The diagnostic performance of indicators for TPE

**Fig. 2:**
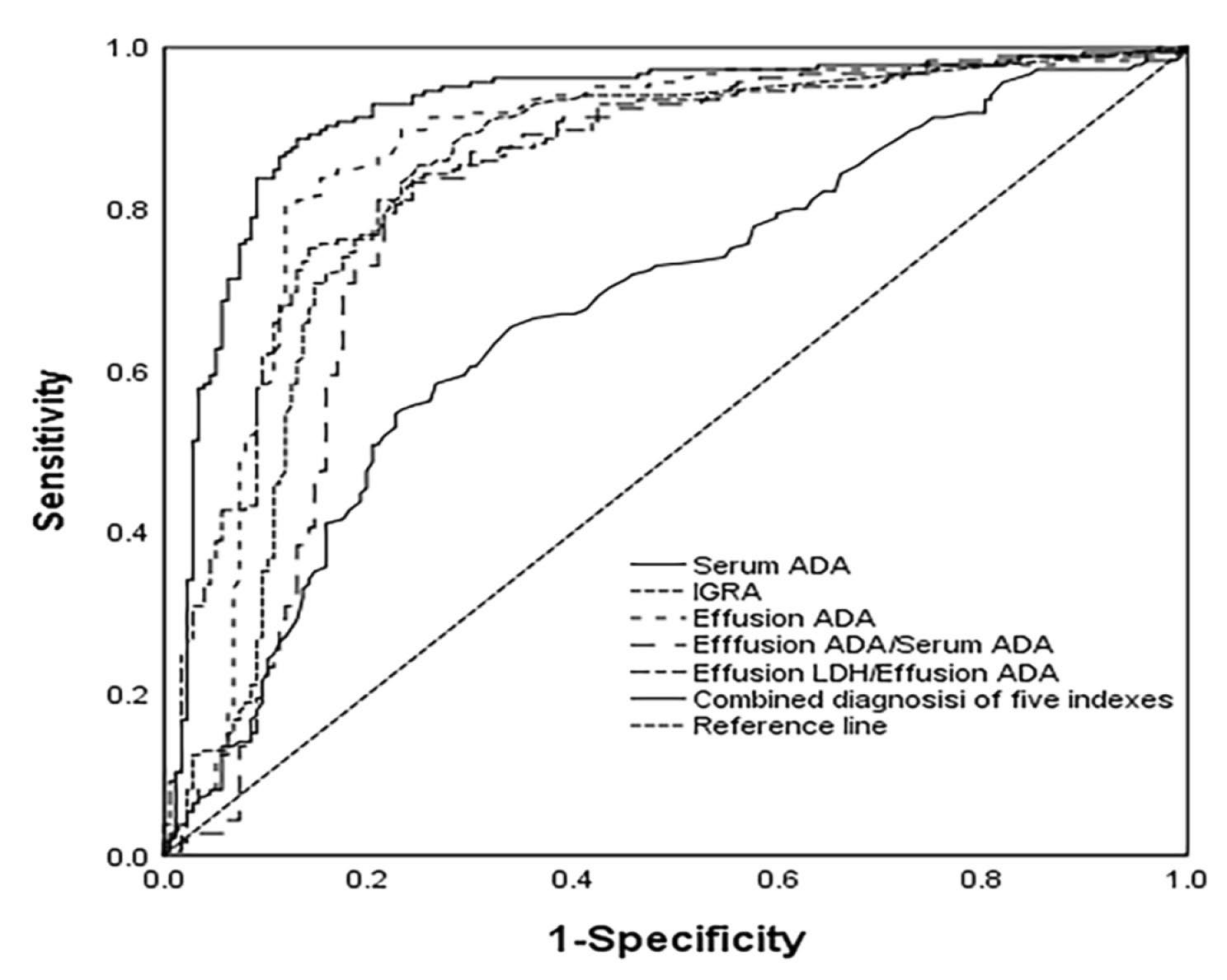
The level of Serum ADA, IGRA, Effusion ADA, Effusion ADA/Serum ADA and Effusion LDH/Effusion ADA and the combined diagnosis of the five indexes are used to discriminate TPE from non-TPE. ROC curve of Serum ADA, IGRA, Effusion ADA, Effusion ADA/Serum ADA and Effusion LDH/Effusion ADA and the combined diagnosis of the five indexes prediction probability discrimination TPE from non-TPE

To distinguish TPE from non-TPE, the diagnostic performance of all indicators was based on ROC. We have defined an AUC greater than 0.65 as a valid marker. The detailed comparative diagnostic reference indicators and their corresponding performance were listed in (Table 3; Fig. 2). The AUCs of effective indexes for differentiating TPE from non-TPE were as follows: serum ADA (0.680, 95% CI 0.624–0.735), IGRA (0.833, 95% CI 0.788–0.878), effusion ADA (0.867, 95% CI 0.825–0.908), effusion ADA/Serum ADA (0.810, 95% CI 0.0.754–0.853), effusion LDH/effusion ADA (0.857, 95% CI 0.0.754–0.853), and 0.919 (0.888–0.951) for combined diagnosis of the five indexes (Table 3; Fig. 2 ).

**Table 3:** Diagnostic performance of the indexes based on ROC in differentiating TPE from non-TPE

Variables	AUC [95%CI]	Sensiti- vity(%)	Specifi- city(%)	PPV (%)	NPV (%)	PLR [(%)	NLR (%)
Serum ADA	0.680 (0.624–0.735)	55.10	76.80	71.30	62.10	2.42	0.58
**IGRA**	**0.833** **(0.788–0.878)**	**85.41**	**75.14**	**78.20**	**83.10**	**3.44**	**0.19**
**Effusion ADA**	**0.867** **(0.825–0.908)**	**80.50**	**88.10**	**87.60**	**81.20**	**6.79**	**0.22**
**Effusion ADA/** **Serum ADA**	**0.810** **(0.754–0.853))**	**82.20**	**76.80**	**78.80**	**80.50**	**3.55**	**0.23**
**Effusion LDH/** **Effusion ADA**	**0.857** **(0.814–0.894)**	**75.14**	**85.90**	**84.80**	**76.80**	**5.32**	**0.29**
**Combined diagnosis of five indexes**	**0.919** **(0.888–0.951)**	**90.30**	**94.50**	**94.35**	**90.27**	**16.42**	**0.10**

Compared to the Serum ADA, IGRA, Effusion ADA, Effusion ADA/Serum ADA and Effusion LDH/Effusion ADA demonstrated a good diagnostic accuracy for TPE in terms of sensitivity (85.41%, 80.50, 82.20, 75.14), and specificity (75.14%, 88.10%, 76.80%, 85.90%), respectively. However, the combined diagnosis of five indexes yielded the highest diagnostic accuracy for TPE with sensitivity of 90.30%, and specificity of 94.50% (Table 3; Fig. 2 ).

## Discussion

For patients with TPE, early diagnosis and timely treatment are essential to keep away from severe complications such as Pleural thickening, empyema, and calcification. However, the early differentiation of TPE from non-TPE (such as PPE and MPE) is still a clinical challenge, despite the availability of various diagnostic methods. In addition, factors such as the low number of bacteria causing the disease, insufficient and unsuitable laboratory samples, and the ineffectiveness of traditional microbiological methods make it difficult for diagnosing TPE.

In this study, we selected 13 variables to differentiate TPE from non-TPE, respectively. These variables comprised of primary clinical and laboratory variables as well as calculated ratios. Finally, we identified the 5 most significant variables for distinguishing TPE from PPE non-TPE, which included Serum ADA, IGRA, Effusion ADA, Effusion ADA/Serum ADA and Effusion LDH/Effusion ADA. These findings demonstrate a strong diagnostic performance. The integration of five commonly used indexes proved to be cost-effective, convenient, and easily accessible in most hospitals.

ADA is a widely studied and recommended biomarker that has shown good performance in diagnosing TPE [17, 18]. A meta-analysis of 2162 citations evaluated the value of Pleural ADA activity in identifying TPE and non-TPE, demonstrating its high sensitivity and specificity (92% and 90%, respectively), including 65 studies with an ADA threshold of 40 ± 4 IU/L [19]. However, a recent study from China showed that the best cutoff value of effusion ADA for TBE was 27U/L with a sensitivity of 81% and a specificity of 78% [20]. Our study also found a similar cutoff value for effusion ADA (25.20 U/L) in differentiating TPE from non-TPE. Therefore, the optimal cutoff values for ADA are still a matter of debate, which may be attributed to variations in disease prevalence rates, sample sizes, different test methods, or the presence of HIV co-infection [17].

The effusion LDH/ADA ratio was also evaluated in differentiating TPE from non-TPE. Blakiston et al. discovered a cutoff value of 15.0 for the effusion LDH/ADA ratio with a high sensitivity and specificity in distinguishing TPE from non-TBE [21]. Another study indicated that the effusion LDH/ADA ratio with other indexes showed a sensitivity and specificity of 80.0% and 87.40% for MPE diagnosis [22]. Additionally, the effusion ADA/LDH ratio showed a sensitivity and specificity of 81.13% and 83.67% at a cutoff value of 14.29, and a high AUC value of 0.888 for the differential diagnosis of TPE from PPE [23]. Similarly, our study showed a cutoff value of 17.49 (sensitivity: 75.14%, specificity: 85.90%) for effusion LDH/ADA to diagnose TPE from non-TPE. More prospective studies need to be carried out to demonstrate our results.

The ESAT-6 and CFP10 of stimulatory antigens used in the IGRA test are specific to MTB and are not affected by BCG and or the immune system, increasing diagnostic specificity. It is also not influenced by non-tuberculous bacilli and BCG on the results, providing excellent value for the diagnosis [24, 25]. Recent studies have shown significant differences in IGRA between TPE and non-TPE groups, such as malignant Pleural effusion, pneumonia, and cirrhosis [26]. Ashutosh N et al. reported a pooled sensitivity and specificity of the blood test for diagnosis of TPE were 0.77 and 0.71, respectively [27]. Furthermore, another study published in 2022 found that the sensitivity and specificity for the blood assays were 0.83 and 0.82 for distinguishing TPE from MPE [30]. Our results showed that the sensitivity and specificity of blood IGRA were 85.41% and 75.14% for differentiating TPE from non-TPE, and the previous studies also supported our findings [27, 28]. Therefore, our study suggests that blood IGRA has potential for serving as a complementary method for diagnosing TPE.

Though IGRA, Effusion ADA, Effusion ADA/Serum ADA and Effusion LDH/Effusion ADA showed a good diagnostic value in distinguishing TPE from non-TPE, a combination of multiple markers to diagnose TPE might be more valuable in clinical practice. Several studies have recommended that the diagnostic value of combinations of two or more markers was greater than any single marker for diagnosing TPE [25, 27, 30]. So, we utilized multiple indicators for the joint diagnosis of TPE in our study, The combination of five indexes, including Serum ADA, IGRA, Effusion ADA, Effusion ADA/Serum ADA and Effusion LDH/Effusion ADA, demonstrated the highest AUC [0.919, 95% CI (0.888–0.951)], which showed 90.30% sensitivity of and 94.50% specificity, outperforming other indicators. Additionally, the combination exhibited a PPV of 94.35%, indicating the likelihood of developing TPE in the patients. PLR and NLR integrated advantages of sensitivity, specificity, PPV, and NPV for disease diagnosis, remaining unaffected by the disease incidence. Therefore, they were relatively independent, Hence, they serve as relatively independent and clinically significant indexes for evaluating diagnostic tests.

When the PLR was greater than 10 or the NLR was less than 0.1, the probability of diagnosing or ruling out the disease significantly increased. The PLR and NLR of the combination of five indexes in diagnosing TPE were 16.42 and 0.10, respectively, indicating a significantly improved diagnostic accuracy of TPE.

In summary, this study evaluated the AUC, sensitivity, specificity PPV, NPV, PLR and NLR of Serum ADA, IGRA, Effusion ADA, Effusion ADA/Serum ADA and Effusion LDH/Effusion ADA for the diagnosis of TPE, used multiple indicators for joint diagnosis of TPE. which provided be reliable and accuracy in distinguishing TPE from non-TPE. Our research included the most common and valuable indexes, which performed better than any single variable alone. Furthermore, the five easily accessible and inexpensive variables routinely tested and acquired in most hospitals. Therefore, our diagnostic indicators could be easily implemented in clinical practice in most hospitals, particularly in primary hospitals.

However, our study has several limitations. Firstly, the study was a single-center retrospective study. To validate our findings, more prospective and multicenter studies with different populations should be conducted. Secondly, our retrospective study only included conventional indexes of serum and PE. It would be beneficial to include newly potential biomarkers, such as interleukin 27 (IL-27), IL-32, tumor necrosis factor-α (TNF-α) and C-X-C motif chemokine ligand 9 (CXCL9), to improve diagnostic accuracy. Thirdly, our study was conducted on Chinese patients, and since the incidence of TB varies from country to country, the results cannot be generalized to patients in other countries. So, multicentric and prospective investigations containing comprehen- sive data was needed to validate our results.

## Conclusion

Combined detection of Pleural effusion of Serum ADA, IGRA, Effusion ADA, Effusion ADA/Serum ADA and Effusion LDH/Effusion ADA can improve the diagnostic efficacy of tuberculous pleurisy.

### Electronic supplementary material

Below is the link to the electronic supplementary material.

Supplementary Material 1

## Data Availability

The datasets used and/or analysed during the current study are available from the corresponding author on reasonable request.

## Abbreviations

ADA: Adenosine deaminase
AIC: Akaike information criterion
ALB: Albumin
AUC: Area under the curve
CI: Confidence intervals
CRP: C-reactive protein
CXCL9: C-X-C motif chemokine ligand 9
ESR: Erythrocyte sedimentation rate
IGRA: Interferon-gamma release assay
IL-27: Interleukin 27
LDH: Lactatedehy drogenas
MPE: Malignant Pleural effusion
NLR: Negative likelihood ratio
NPV: Negative predictive value
ORs: Odds ratios
PE: Pleural effusion
PLR: Positive likelihood ratio
PPE: Parapneumonic Pleural effusion
PPV: Positive predictive
ROC: Receiver operating characteristic
TB: Tuberculosis
TBP: Tuberculous pleurisy
TNF-α: Tumor necrosis factor-α. TP:total protein
TPE: Tuberculous Pleural effusion
