# Supplementary material for: Biomarkers for distinguishing tuberculous pleural effusion from non-tuberculosis effusion: a retrospective study
Source: BMC Infect Dis. 2023 Nov 8;23:771. doi: 10.1186/s12879-023-08781-0 (PMC10633909; doi:10.1186/s12879-023-08781-0)
Supplement: Supplementary file 1 — Supplementary Material 1 [file 12879_2023_8781_MOESM1_ESM.docx]

| **Table S1** Univariate logistic regression analysis of the clinical characteristics for discriminating TPE from PPE | | | | |
| --- | --- | --- | --- | --- |
| Variables | P value (Mann- Whi tney U test) | Cut-off | AUC(95%CI) | Univariate analysis OR (95% CI) P value |
| Age (years) | 0.000 | 43.00 | 0.667(0.606-0.729) | 0.968(0.956–0.981) 0.000 |
| IGRA(pg/ml) | 0.000 | 55.00 | 0.845(0.794-0.897) | 1.014(1.009–01.018) 0.000 |
| CRP(mg/L) | 0.367 | 101.71 | 0.558(0.485-0.631) | 0.991(0.986-0.996)0.001 |
| ESR(mm) | 0.567 | 36.5 | 0.533(0.463-0.604) | 1.011(0.999-1.023)0.068 |
| Serum TP(g/l) | 0.006 | 67.80 | 0.597(0.530-0.663) | 1.035(1.009-1.062)0.008 |
| Serum ALB(g/l) | 0.003 | 34.15 | 0.604(0.534-0.674) | 1.070(1.015-1.127)0.011 |
| Serum ADA(U/l) | 0.000 | 11.65 | 0.652(0.586-0.718) | 1.117(1.049–1.190) 0.001 |
| Serum LDH(U/L) | 0.049 | 169.50 | 0.570(0.495-0.646) | 0.999(0.998-1.001)0.399 |
| Effusion TP(g/l) | 0.000 | 44.35 | 0.670(0.603-0.737) | 1.061(1.031-1,092)0.000 |
| Effusion ALB (g/l) | 0.000 | 26.05 | 0.682(0.618-0.747) | 1.111 (1.060–1.165) 0.000 |
| Effusion ADA(U/L) | 0.000 | 25.15 | 0.835(0.779-0.891) | 1.070(1.049-1.092)0.000 |
| Effusion LDH(U/L) | 0.000 | 231.00 | 0.624(0.548-0.700) | 0.467(1.000-1.000)0.467 |
| Effusion ADA/ Serum ADA | 0.000 | 1.97 | 0.756(0.689-0.823) | 1.446(1.213-1.724)0.000 |
| Effusion LDH/ Effusion ADA | 0.000 | 17.49 | 0.806(0.753-0.859) | 2.175E+15(7.979E+10-5.928E+20)0.000 |
| Serum IGRA/ EffusionADA | 0.000 | 0.55 | 0.748(0.684-0.811) | 1.077(1.017-1.141)0.012 |

| **Table S2** Univariate logistic regression analysis of the clinical characteristics for discriminating TPE from MPE | | | | |
| --- | --- | --- | --- | --- |
| Variables | P value (Mann- Whi tney U test) | Cut-off | AUC(95%CI) | Univariate analysis OR  (95%CI) P value |
| Age (years) | 0.000 | 52.50 | 0.745(0.684-0.807) | 0.952(0.936-0.969)0.000 |
| IGRA(pg/ml) | 0.000 | 11.33 | 0.812(0.742-0.882) | 1.012(1.004-1.019)0.003 |
| CRP(mg/L) | 0.017 | 45.00 | 0.682(0.594-0.770) | 1.039(1.025-1.054)0.000 |
| ESR(mm) | 0.272 | 67.85 | 0.780(0.700-0.860) | 1.032(1.005-1.060)0.019 |
| Serum TP(g/l) | 0.006 | 33.55 | 0.653(0.565-0.742) | 0.963(0.916-1.012)0.136 |
| Serum ALB(g/l) | 0.003 | 9.55 | 0.544(0.461-0.627) | 1.181(1.089-1.281)0.000 |
| Serum ADA(U/l) | 0.000 | 196.00 | 0.679(0.592-0.766) | 0.995(0.991-0.998)0.004 |
| Serum LDH(U/L) | 0.032 | 48.90 | 0.561（0.466-0.657） | 1.026(0.999-1.053)0.055 |
| Effusion TP(g/l) | 0.000 | 20.95 | 0.662（0.574-0.750） | 1.066(1.019-1.115)0.006 |
| Effusion ALB (g/l) | 0.006 | 19.45 | 0.640（0.550-0.731） | 1.181(1.089-1.281)0.000 |
| Effusion ADA(U/L) | 0.000 | 402.50 | 0.911（0.8613-0.960） | 1.646(1,313-1.735)0.000 |
| Effusion LDH(U/L) | 0.033 | 1.63 | 0.781（0.704-0.858） | 1.039(1.011-1.067)0.000 |
| Effusion LDH / Serum LDH | 0.000 | 1.80 | 0.654（0.575-0.733） | 1.026(0.927-1.135)0.622 |
| Effusion ADA/ Serum ADA | 0.000 | 2.07 | 0.872（0.816-0.928） | 2.646(1,978-3.540)0.000 |
| Effusion LDH/  Effusion ADA | 0.000 | 21.39 | 0.929（0.894-0.964） | 5.505E+37(4.731E+27- 6.405E+47)0.000 |

| Table S3 Univariate logistic regression analysis of the clinical characteristics for discriminating TPE from non-TPE | | | | |
| --- | --- | --- | --- | --- |
| Variables | Pvalue (M-W U test) | Cut-off | AUC(95%CI) | Univariate analysis OR (95% CI) P value |
| Age (years) | <0.05 | 50.5 | 0.699(0.646-0.753) | 0.960(0.948–0.972) <0.001 |
| IGRA(pg/ml) | <0.05 | 26.5 | 0.833(0.788-0.878) | 1.010(1.007–1.012) <0.001 |
| CRP(mg/L) | 0.138 | 101.71 | 0.434(0.395-0.497) | 0.991(0.986-0.996)0.001 |
| ESR(mm) | <0.05 | 36.5 | 0.622(0.544-0.681) | 1.015(1.007-1.024)0.001 |
| Serum TP(g/l) | <0.05 | 68.64 | 0.660(0.603-0.716) | 1.037(1.014-1.060)0.001 |
| Serum ALB(g/l) | 0.158 | 34.15 | 0.454(0.397-0.513) | 1.070(1.015-1.127)0.011 |
| Serum ADA(U/l) | <0.05 | 10.95 | 0.680(0.624-0.735) | 1.131(1.072–1.194) <0.001 |
| Serum LDH(U/L) | 0.853 | 169.50 | 0.474(0.413-0.523) | 0.999(0.998-1.001)0.399 |
| Effusion TP(g/l) | <0.05 | 44.35 | 0.660(0.603-0.716) | 1.038(1.015-1,061) <0.001 |
| Effusion ALB (g/l) | <0.05 | 26.05 | 0.653(0.596-0.709) | 1.084 (1.044–1.126)<0.001 |
| Effusion ADA(U/L) | <0.05 | 25.2 | 0.867(0.825-0.908) | 1.076(1.057-1.095) <0.001 |
| Effusion LDH(U/L) | <0.05 | 255.5 | 0.601(0.541-0.662) | 1.000(1.000-1.000)0.275 |
| Effusion ADA/ Serum ADA | <0.05 | 2.07 | 0.810(0.754-0.853) | 1.546(1.327-1.802) <0.001 |
| Effusion LDH/ Effusion ADA | <0.05 | 17.49 | 0.857(0.814-0.894) | 0.922(0.903-0.941) <0.001 |
| Serum IGRA/ EffusionADA | <0.05 | 0.64 | 0.722(0.684-0.811) | 0.987(0.952-1.023)0.482 |
| Effusion LDH/  Serum LDH | <0.05 | 1.65 | 0.616(0.558-0.675 | 0.998(0,.982-1.014)0.773 |
